# Supplementary material for: The interactome of intact mitochondria by cross-linking mass spectrometry provides evidence for coexisting respiratory supercomplexes
Source: Mol Cell Proteomics. 2017 Dec 8;17(2):216–32. doi: 10.1074/mcp.RA117.000470 (PMC5795388; doi:10.1074/mcp.RA117.000470)
Supplement: Supplemental Data [file supp_RA117.000470_133922_0_supp_23978_fzffwf.zip › spectra_annotation/mito_DR_spectra_annotation/333-1-11-1-8-1.pdf]

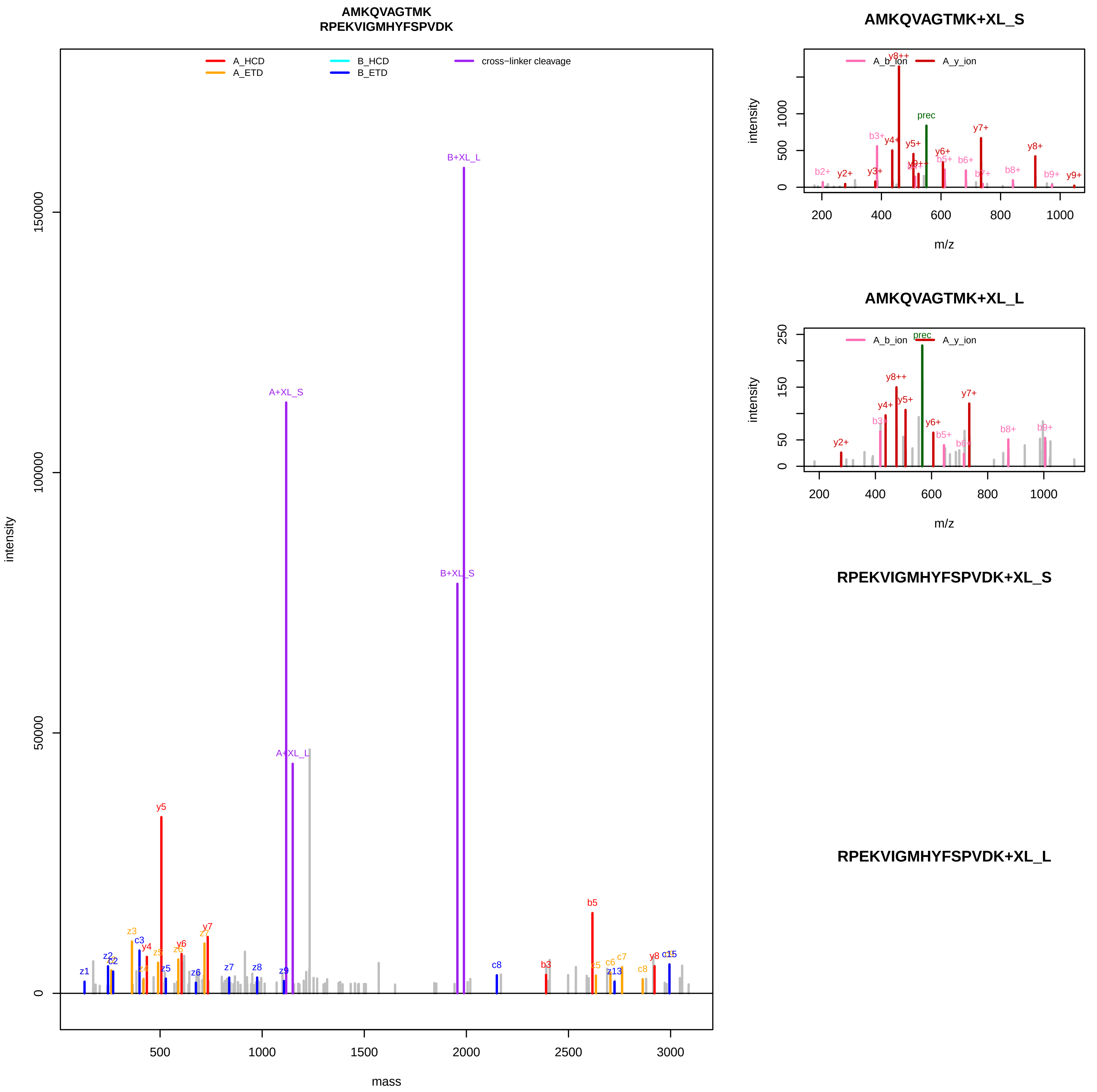
AMKQVAGTMK
RPEKVIGMHYFSPVDK
A_HCD
A_ETD
B_HCD
B_ETD
cross−linker cleavage
B+XL_L
A+XL_S
B+XL_S
A+XL_L
y5
b5
y7
y6
y4
z3
c3
z2
c2
z1
z5
z6
z7
z8
z9
c8
b3
c5
c6
c7
z13
c8
y8
c15
intensity
mass
0
50000
100000
150000
500
1000
1500
2000
2500
3000
AMKQVAGTMK+XL_S
A_b_ion
A_y_ion
y8++
prec
b3+
y4+
y5+
y7+
y6+
b5+
b6+
y8+
b2+
y2+
y3+
b8+
b9+
y9+
intensity
m/z
0
500
1000
200
400
600
800
1000
AMKQVAGTMK+XL_L
A_b_ion
A_y_ion
prec
y8++
y4+
y5+
b3+
y7+
y6+
b5+
b8+
b9+
y2+
intensity
m/z
0
50
150
250
200
400
600
800
1000
RPEKVIGMHYFSPVDK+XL_S
RPEKVIGMHYFSPVDK+XL_L
